# Supplementary material for: Generating Heterokaryotic Cells via Bacterial Cell-Cell Fusion
Source: Microbiol Spectr. 2022 Jul 14;10(4):e01693-22. doi: 10.1128/spectrum.01693-22 (PMC9430406; doi:10.1128/spectrum.01693-22)
Supplement: Supplemental file 1 — Supplemental material. Download spectrum.01693-22-s0002.pdf, PDF file, 2.3 MB [file spectrum.01693-22-s0002.pdf]

Supplementary material for

Generating heterokaryotic cells via targeted cell-cell fusion in wall-deficient bacteria

Shraddha Shitut*[1,2,3], Meng-Jie Shen[2], Bart Claushuis[3], Rico J. E. Derks[4], Martin Giera[4], Daniel Rozen[3], Dennis Claessen*[3], Alexander Kros*[2]

[1] Origins Centre, Groningen, the Netherlands
[2] Dept. Supramolecular & Biomaterials chemistry, Leiden Institute of Chemistry, Leiden University, the Netherlands
[3] Institute of Biology, Leiden University, the Netherlands
[4] Center for Proteomics and Metabolomics, Leiden University Medical Center, the Netherlands

Correspondence to: shraddha.shitut@gmail.com (SS), d.claessen@biology.leidenuniv.nl (DC), a.kros@chem.leidenuniv.nl (AK)

Description	Page no.
Figures	2
Tables	10
Methods	11

Figures

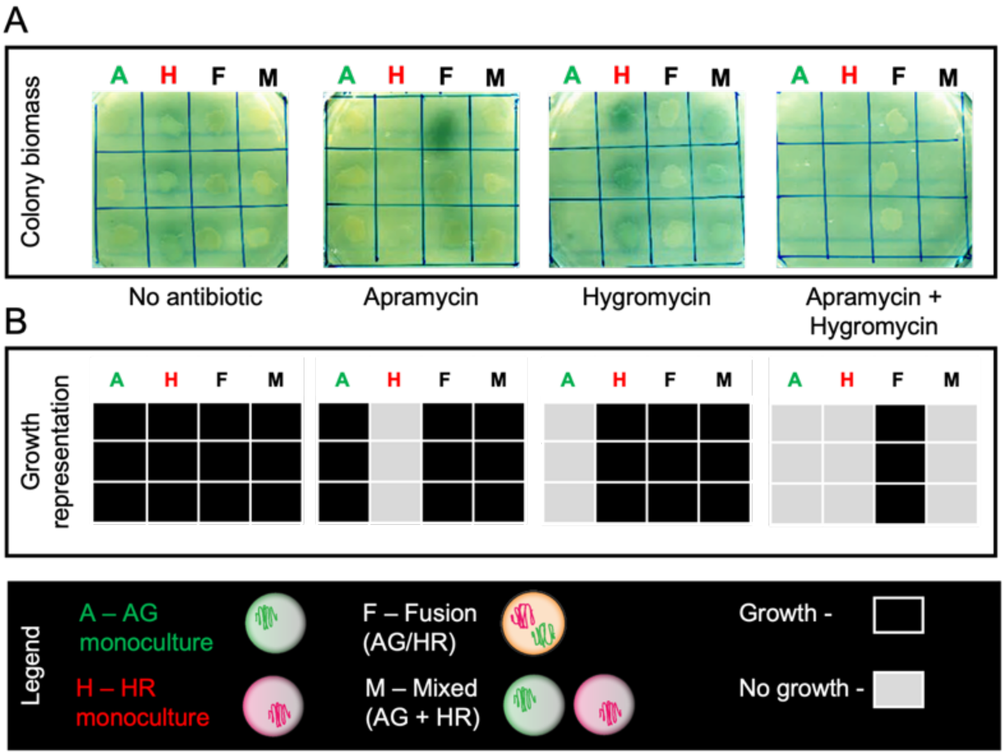

Fig S1. Fusion is a prerequisite for growth on double antibiotic selection. (**A**) Strains AG (A), HR (H), Fusant (F) and Mixed (M, see legend panel) plated on media with different selection (No antibiotic, only apramycin, only hygromycin and both apramycin and hygromycin). All strains grow under no antibiotic selection. Visible growth of AG on apramycin and HR on hygromycin. Fusant grows on all selection environments whereas mixed does not grow on apramycin + hygromycin selection. (**B**) Growth from biomass plates represented as black and grey boxes for easy interpretation followed by the legend.

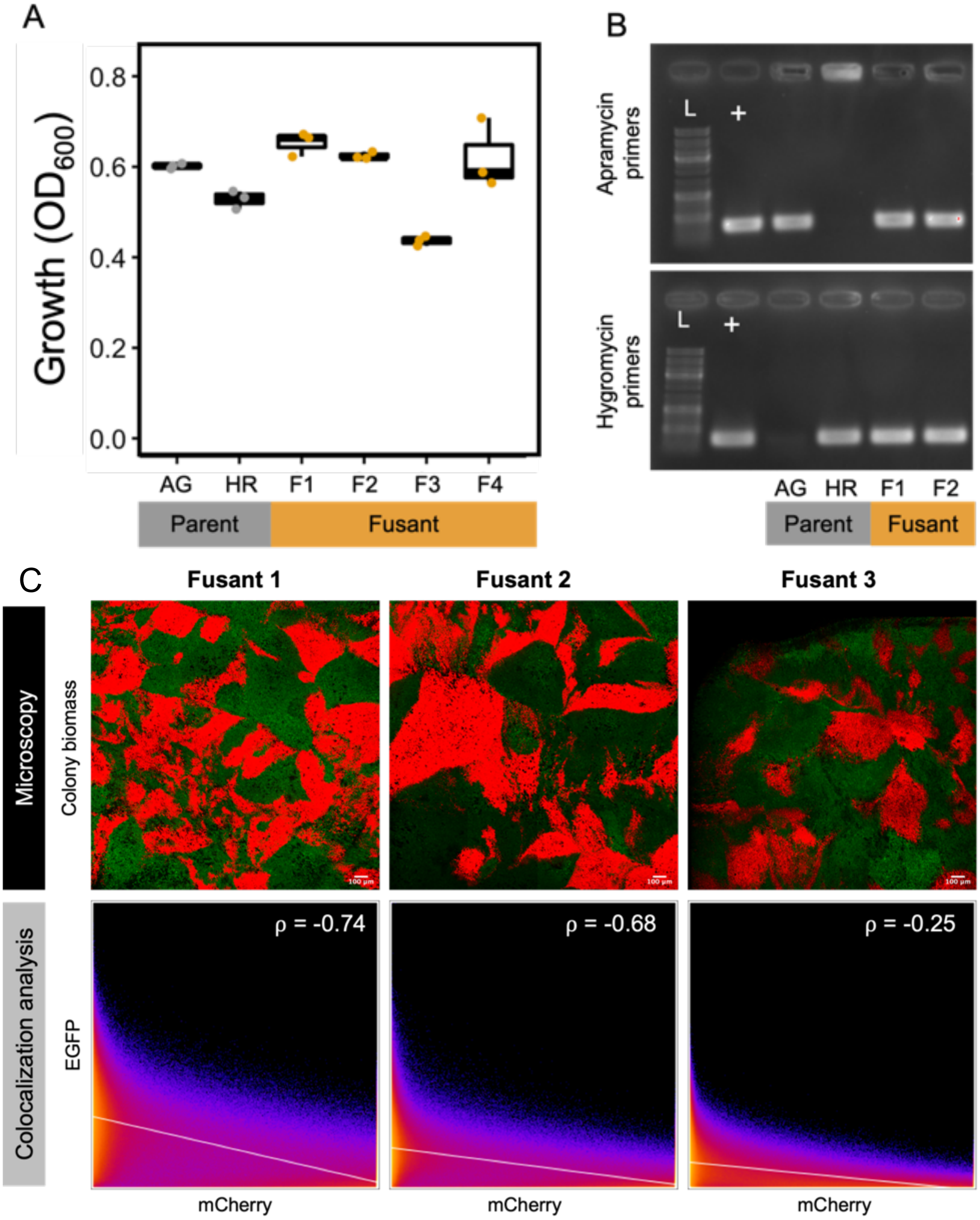
A
Growth (OD_{600})
0.8
0.6
0.4
0.2
0.0
AG
HR
F1
F2
F3
F4
Parent
Fusant
B
Apramycin primers
Hygromycin primers
L
+
AG
HR
F1
F2
Parent
Fusant
C
Fusant 1
Fusant 2
Fusant 3
Microscopy
Colony biomass
100 µm
Colocalization analysis
EGFP
ρ = -0.74
ρ = -0.68
ρ = -0.25
mCherry
mCherry
mCherry

Fig. S2. Growth and genomic confirmation of fused strains. (**A**) All cultures: parent (grey) and fusant (yellow) were grown in liquid media under selection (apramycin for AG, hygromycin for HR and both for the fusant) for 4 days at 30ºC to confirm viability after fusion. Optical density measured at 600 nm indicates growth of all populations. (**B**) PCR confirmation of the fusant containing both genetic markers. Genomic DNA was used from 3-day old cultures along with primers for regions of the apramycin (top panel) and hygromycin (bottom panel) resistance gene. First lane after the ladder (L) has a positive control for each primer with purified amplicons (+) as template. Monoculture of AG shows amplification with only apramycin primers whereas monoculture of HR shows amplification only with hygromycin primers. Fusants show amplification for both apramycin and hygromycin. (**C**) Segregation of fusant colonies under no selection. Individual colonies picked after fusion were grown on media without antibiotics. Fluorescence microscopy of biomass (top panel) shows the presence of green and red sectors/regions when no selection pressure is applied indicating a segregation of both chromosome types in daughter cells. Each image was subjected to colocalization analysis of intensities of each pixel in the two channels (EGFP and mCherry) to quantify the overlap of green and red pixels. A negative correlation (Spearman's rho) was observed for each biomass indicating no overlap between green and red pixels in the image. Without the selection pressure of antibiotics cells lose either one chromosome leading to only green or only red patches. Scale bar = 100 μm.

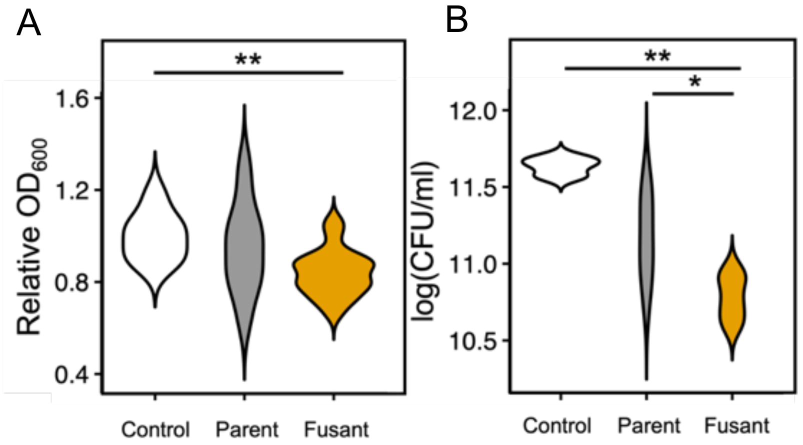

Fig. S3. Antimicrobial activity of fused strains. (A) *E. coli* JM109 indicator strain was grown in different conditions; monoculture (white, control), cocultured with parent AG and HR (grey, parent) and cocultured with fused strains (yellow, fusant) to test the antimicrobial activity of the L-form strains. Cell density (OD_{600}) of *E.coli* was measured at 48 h and normalized by the OD_{600} of the partner strain to allow comparison. Significantly lower relative OD was obtained in coculture with fusant strains (Dunnets pairwise comparison, p=0.0083, n=8). (**B**) The antimicrobial effect of L-forms on *E. coli* was also quantified through cell counts over 48 h. Shown here is the $\log_{10}$ transformed values of colony forming units per ml (CFU/ml). *E. coli* showed a decreased growth when in coculture with fusants compared to the control (Dunnets pairwise comparison, p=0.0033, n=8) and the parent strains (Dunnets pairwise comparison, p=0.045, n=8).

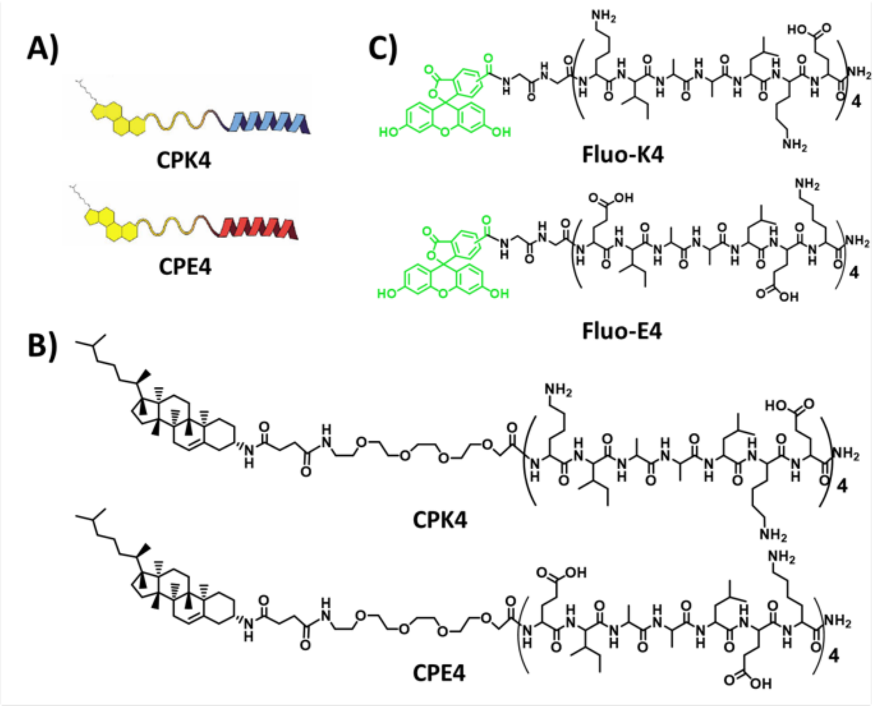

Fig. S4. Schematic representation (**A**) and chemical structure (**B**) of CPK_4 and CPE_4. (**C**) Chemocal structure of fluo-K_4 and fluo-E_4.

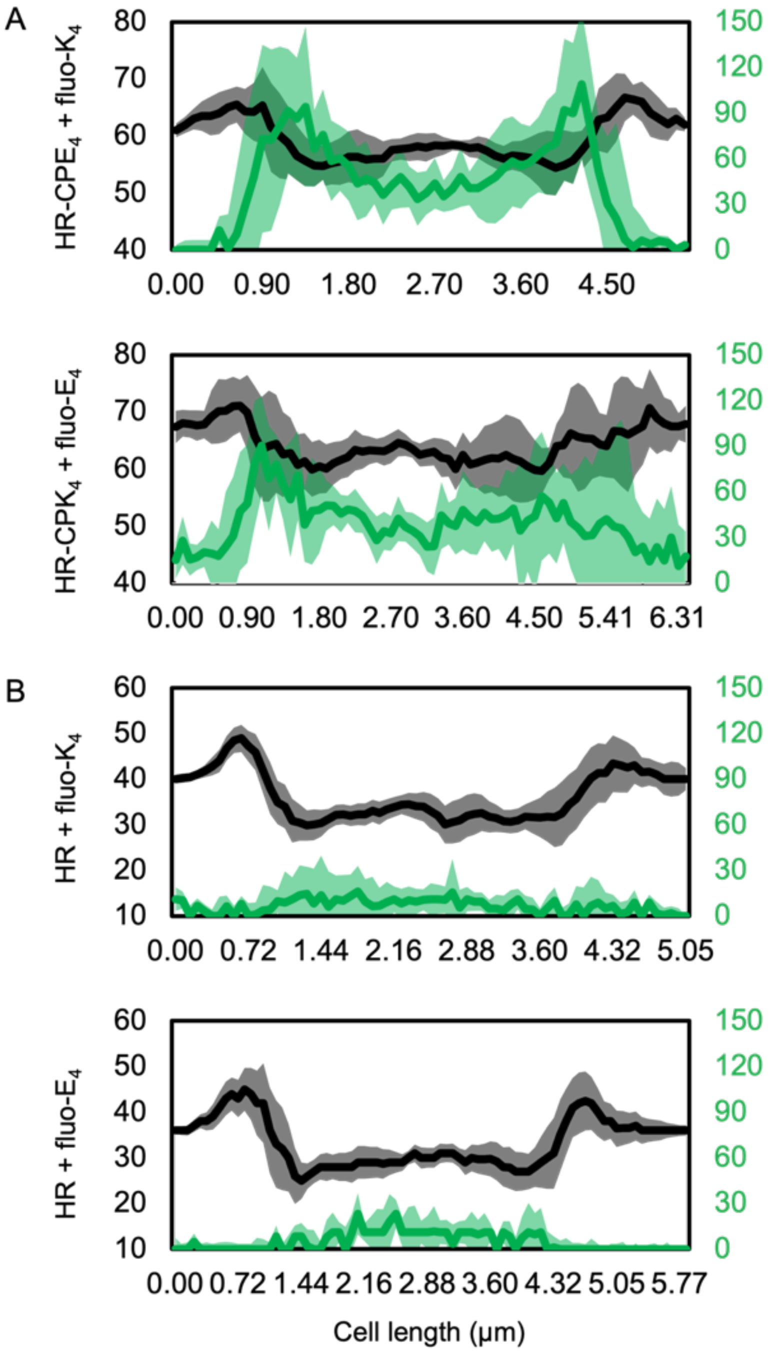

Fig. S5. Coiled coil peptides localize on the membrane. (**A**) Image analysis of the membrane labelled L-forms (HR) was done to obtain intensity profiles across the cell (n=10). Shown is the median ± SD values for the cell in brightfield (black, left y axis) and the eGFP (green, right y axis) for the different treatments (same as in figure 3). The profiles indicate peaks of fluorescence (green) coinciding with the boundary of cells also seen as peaks (black). The fluorescence intensities decrease in the central region of the cell. (**B**) The controls where no coiled coil lipopeptide (CPE_4 or CPK_4) was added to the cells show no peaks of fluorescence (green) across the cell profile (black).

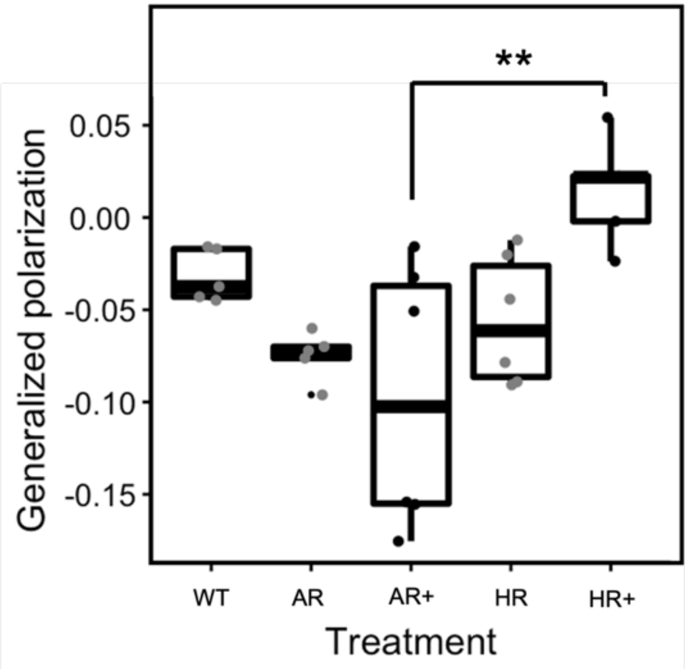

Fig. S6. Antibiotic alters membrane fluidity. Strains (WT, AR and HR) were grown in LPB media in the absence or presence (+) of the selective antibiotic (apramycin for AR and hygromycin for HR) for 2 days and tested for membrane fluidity using the Laurdan assay. WT, AR and HR have similar basal levels of fluidity around -0.05. Apramycin treatment increases the fluidity of the AR strain whereas hygromycin treatment decreases fluidity of the HR strain. These differences in GP value between the AR+ and HR+ conditions (One-way ANOVA, F=5.85, p=0.002 followed by Tukey's pairwise comparison) are like that observed in figure 6 in the control treatment of AR and HR strains.

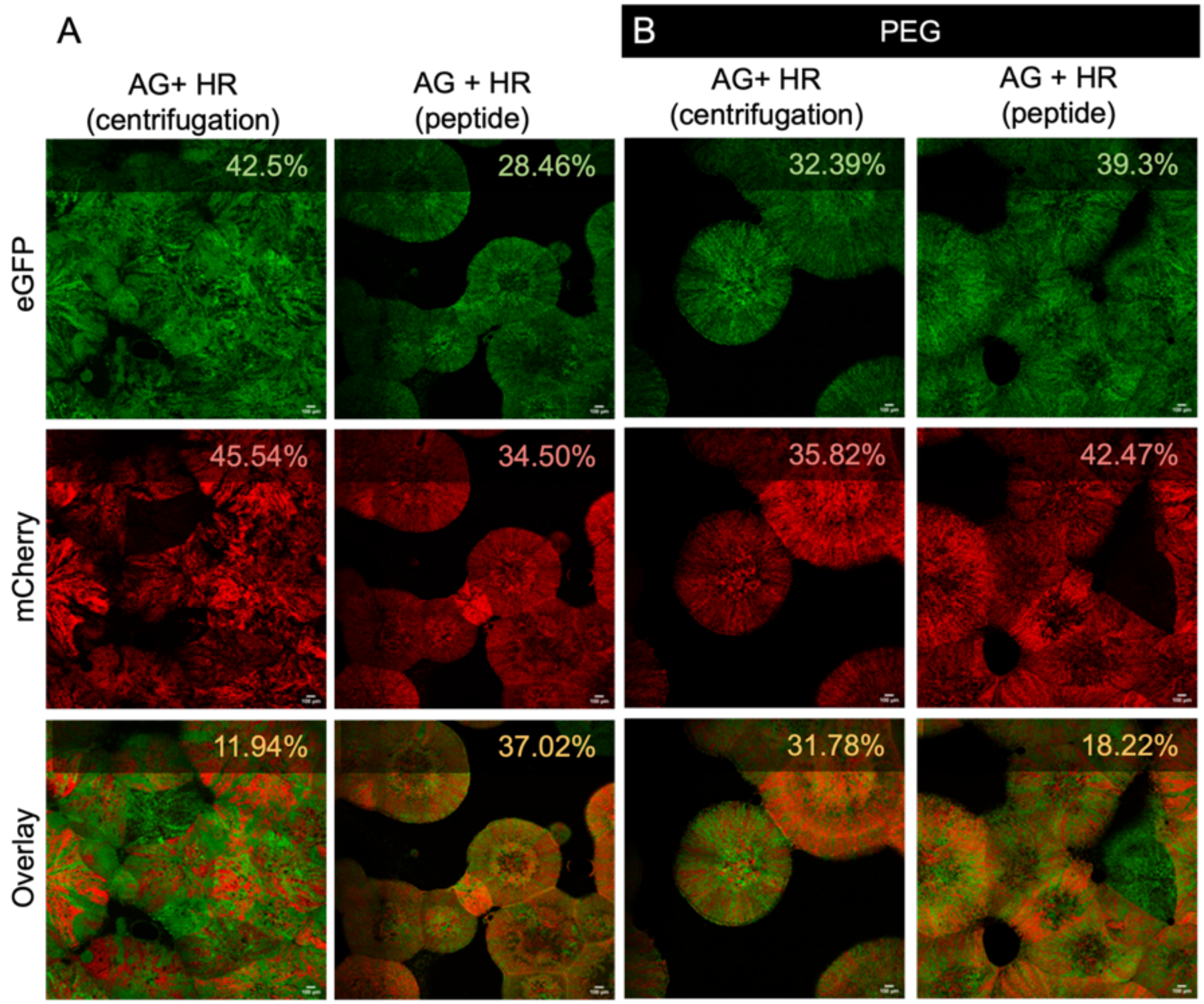

Fig. S7. Microscopy of fused colony. (A) Strains AG and HR were fused using centrifugation and peptides followed by selection of colonies on double antibiotic medium. Whole colonies were imaged for measuring expression of fluorescence reporters EGFP (top row) and mCherry (middle row). The percent of fluorescence is indicated in the top right corner of each image and was calculated using ImageJ/Fiji. (**B**) Fusion of strains using centrifugation or peptides in combination with PEG resulted in more colonies on

double selection medium. This colonies were imaged, and fluorescence percent was calculated. Scale bar = 100 µm.

Table S1. Primer sequences used to amplify gap1-mCherry for the pRed2 plasmid construction.

**Primer**	**Sequence 5'-3'**
Gap-FW-XbaI	GATTACTCTAGACCGAGGGCTTCGAGAC
mCherry-RV-XbaI	TAAGCATCTAGACTAGCCGCCACACTTGTAC
Apramycin-FW	GCAATACGAATGGCGAAAAG
Apramycin-RV	AGATGATCTGCTCTGCCTG
Hygromycin-FW	ACCGTGCTCACCCCCCATTC
Hygromycin-RV	CCGGAAGGCGTTGAGATGCA

Table S2. Calculated mass and found mass via LC-MS of CPK_4 and CPE_4.

**Peptide**	**Mass (calcd.) / Da**	**Mass (found) / Da**
**CPK_4**	$[M + 2H^+]^{2+}$ 1867.9	1866.4
	$[M + 3H^+]^{3+}$ 1245.2	1244.3
**CPE_4**	$[M + 2H^+]^{2+}$ 1869.7	1868.8
	$[M + 3H^+]^{3+}$ 1246.5	1245.4
**Fluo-K_4**	$[M + 2H^+]^{2+}$ 1876.5	1874.0
	$[M + 3H^+]^{3+}$ 1251.3	1249.3
**Fluo-E_4**	$[M + 2H^+]^{2+}$ 1877.4	1875.9
	$[M + 3H^+]^{3+}$ 1251.6	1250.6

Supplementary methods

Lipid extraction and analysis

Cultures of the wildtype L-form were grown for different time periods (1, 3, 5 and 7 days). These were centrifuged and resuspended in P-buffer prior to membrane lipidomics. Lipids where extracted using a modified MTBE protocol (Matyash et al. 2008). In short, 600 µL MTBE and 150 µL methanol were added to the thawed bacteria samples. Samples where briefly vortexed, ultra-sonicated for 10 minutes and shaken at room temperature for 30 minutes. Next, 300 µL water was added and the samples where centrifuged for 5 minutes at 18213 ×g at 20 ˚C. After centrifugation, the upper layer was collected and transferred to a glass vial. The extraction was repeated by adding 300 µL MTBE and 100 µL methanol. Samples where briefly vortexed and shaken at room temperature for 5 minutes. Next, 100 µL water was added and the samples where centrifuged for 5 minutes at 18213 ×g at 20 ˚C. After centrifugation, the upper layer was collected, and the organic extracts combined. Samples where dried under a gentle stream of nitrogen. After drying samples were reconstituted in 100 µL 2-propanol. After briefly vortexing and ultra-sonication for 5 minutes, 100 µL water was added. Samples were transferred to microvial inserts for analysis

Lipidomic analysis of bacteria lipid extracts was performed using a LC-MS/MS based lipid profiling method (PMID: 31972163 DOI: 10.1016/j.bbamem.2020.183200). A Shimadzu Nexera X2 (consisting of two LC30AD pumps, a SIL30AC autosampler, a CTO20AC column oven and a CBM20A controller) (Shimadzu, 's Hertogenbosch, The Netherlands) was used to deliver a gradient of water:acetonitrile 80:20 (eluent A) and water:2-propanol:acetonitrile 1:90:9 (eluent B). Both eluents contained 5 mM ammonium formate and 0.05% formic acid. The applied gradient, with a column flow of 300 µL/min, was as follows: 0 min 40% B, 10 min 100% B, 12 min 100% B. A Phenomenex Kinetex C18, 2.7 µm particles, 50 × 2.1 mm (Phenomenex, Utrecht, The Netherlands) was used as column with a Phenomenex SecurityGuard Ultra C8, 2.7 µm, 5 × 2.1 mm cartridge (Phenomenex, Utrecht, The Netherlands) as guard column. The column was kept at 50 ˚C. The injection volume was 10 µL.

The MS was a Sciex TripleTOF 6600 (AB Sciex Netherlands B.V., Nieuwerkerk aan den Ijssel, The Netherlands) operated in positive (ESI+) and negative (ESI-) ESI mode, with the following conditions: ion source gas 1 45 psi, ion source gas 2 50 psi, curtain gas 35 psi, temperature 350˚C, acquisition range *m/z* 100-1800, ion spray Voltage 5500 V (ESI+) and -4500 V (ESI-), declustering potential 80 V (ESI+) and -80 V (ESI-). An information dependent acquisition (IDA) method was used to identify lipids, with the following conditions for MS analysis: collision energy ±10, acquisition time 250 ms and for MS/MS analysis: collision energy ±45, collision energy spread 25, ion release delay 30, ion release width 14, acquisition time 40 ms. The IDA switching criteria were set as follows: for ions greater than *m/z* 300, which exceed 200 cps, exclude former target for 2 s, exclude isotopes within 1.5 Da, max. candidate ions 20.

Before data analysis, raw MS data files were converted with the Reifycs Abf Converter (v1.1) to the Abf file format. MS-DIAL (v4.20), with the FiehnO (VS68) database was used to align the data and identify the different lipids (Tsugawa et al. 2015; 2019; 2020). Further processing of the data was done with R version 4.0.2 (R Core Team 2014).
The relative abundance of specific lipid class *vs* total relative abundance was used to roughly compare the ratio of each lipid class. The lipids have been sorted into saturated and unsaturated lipids classes. Also, the lipids have been sorted based on head groups (DG, TG, PE, PI) and the ratio of each class have been calculated.

Antimicrobial activity assay
The antimicrobial effect of L-form strains (parental and fusant) was tested against *Escherichia coli* JM109 which is a susceptible strain. A 24 well Transwell® plate (Cornig) with permeable supports was used for coculturing the L-forms with *E. coli* in the same medium. The permeable supports holding the *E. coli* cells were lined with a 0.4 µm filter which blocks the transfer of cells but allows that of small molecules. The outer well consisted of 1.2 mL 4-day old cultures of L-forms whereas the supports contained 300 µL of *E. coli* cultures in LPB medium. *E. coli* was precultured in LB medium to exponential phase (0.4 OD_{600}), centrifuged and resuspended in LPB. Monoculture controls of all strains were maintained in the same condition and volume. The plate was incubated at 30°C 100 rpm for 48 hours. 100 µL of each culture was taken for density measurement and diluted with 100 µL LPB. Additionally, 20 µL culture was used for serial dilution and plating on LB agar to quantify change in cell number.

Lipopeptide preparation and treatment
Peptide K_4 and E_4 were synthesized on a CEM Liberty Blue microwave-assisted peptide synthesizer using Fmoc chemistry. 20% piperidine in DMF was used as the deprotection agent. During coupling, DIC was applied as the activator and Oxyma as the base. All peptides were synthesized on a Tentagel S RAM resin (0.22 mmol/g). The resin was swelling for at least 15min before synthesis started. For the coupling, 5 equivalents of amino acids (2.5 mL in DMF), DIC (1 mL in DMF) and Oxyma (0.5 mL in DMF) were added to the resin in the reaction vessel and were heated to 90 ℃ for 4 minutes to facilitate the reaction. For deprotection, 20% of piperidine (4 mL in DMF) was used and heated to 90℃ for 1 minute. Between deprotection and peptide coupling, the resin has been washed three times using DMF. After peptide synthesis, a polyethyleneglycol $(PEG)_4$ linker and cholesterol were coupled manually to the peptide on-resin. 0.1 mmol of each peptide was reacted with 0.2 mmol N_3-PEG_4-COOH by adding 0.4 mmol HCTU and 0.6 mmol DIPEA in 3 mL DMF. The reaction was performed at room temperature for 5 hours. After thorough washing, 3 mL of 0.5 mmol trimethylphosphine in a 1,4-dioxane:H_2O (6:1) mixture was added to the resin to reduce the azide group to an amine (overnight reaction). After reduction, the peptide was reacted with cholesteryl hemisuccinate (0.3 mmol) in DMF by

adding 0.4 mmol HCTU and 0.6 mmol DIPEA. The reaction was performed at room temperature for 3 hours. Lipopeptides were cleaved from the resin using 3 mL of a TFA:triisopropylsilane (97.5:2.5%) mixture and shaking for 50 min. After cleavage, the crude lipopeptides were precipitated by pouring into 45 mL of -20 ℃ diethyl ether:n-hexane (1:1) and isolated by centrifugation. The pellet of the lipopeptides was redissolved by adding 20 mL H_2O containing 10% acetonitrile and freeze-dried to yield a white powder. Lipopeptides were purified with reversed-phase HPLC on a Shimazu system with two LC-8A pumps and an SPD-20A UV-Vis detector, equipped with a Vydac C4 column (22 mm diameter, 250 mm length, 10 µm particle size). CPK4 was purified using a linear gradient from 20 to 65 % acetonitrile in water (with 0.1% TFA) with a 12 mL/min flow rate over 36 mins. CPE4 was purified using a linear gradient from 20 to 75 % acetonitrile in water (with 0.1% TFA) with a 12 mL/min flow rate over 36 mins. After HPLC purification, all peptides were lyophilized and yielded white powders.

For the fluo-K_4 and fluo-E_4 synthesis, two additional glycine residues were coupled to the N-terminus of the peptides on resin, before the dye was manually coupled by adding 3 mL DMF containing 0.2 mmol 5(6)-carboxyfluorescein, 0.4 mmol HCTU and 0.6 mmol DIPEA. The reaction was left at room temperature overnight. The fluo-K_4 and fluo-E_4 were cleaved from the resin using 3 mL of a TFA:triisopropylsilane:H_2O (97.5:2.5%) mixture and shaking for 1.5 hours. After cleavage, the crude lipopeptides were precipitated by pouring into 45 mL of -20 ℃ diethyl ether and isolated by centrifugation. The pellet of the lipopeptides was redissolved by adding 20 mL H_2O containing 10% acetonitrile and freeze-dried to yield a white powder. Fluo-K_4 and fluo-E_4 were purified using the same HPLC described above equipped with a Kinetix Evo C18 column (21.2 mm diameter, 150 mm length, 5 µm particle size). For the fluo-K4, a linear gradient from 20 to 45% acetonitrile in water (with 0.1% TFA) with a 12 mL/min flow rate over 28 mins was used. For fluo-E4, linear gradient from 20 to 55% was used. After HPLC purification, all peptides were lyophilized and yielded orange powders. The purity of all peptides were determined by LC-MS (supplementary table 2). The structure of all peptides used in this study can be found in supplementary figure 3. Treatment of cultures with different peptides was done by adding externally to cells suspended in P-buffer and incubating for 30 minutes at 30°C 100 rpm. Excess peptide was washed by centrifugation.

SI References

Matyash, Vitali, Gerhard Liebisch, Teymuras V. Kurzchalia, Andrej Shevchenko, and Dominik Schwudke. 2008. “Lipid Extraction by Methyl-Tert-Butyl Ether for High-Throughput Lipidomics.” *Journal of Lipid Research* 49 (5): 1137–46.

Legend for supplementary movie

S1 – Timelapse microscopy of fusant culture grown in liquid media containing both antibiotics over 16 hours. Screenshots from this movie have been used in main Figure 3 to show growth ad viability after fusion. Cells can be seen extending membrane and giving rise to spherical daughter cells expressing both fluorescent markers. Scale bar = 5 μm.

S2 - Timelapse imaging of more fusant cells showing growth and division. Observed are membrane extensions along with spherical daughter cells expressing both EGFP and mCherry. Scale bar = 5 μm.

S3 - Timelapse imaging of cell undergoing lysis to show the sudden disappearance of both fluorescent signals. Scale bar = 5 μm.
